# Supplementary material for: An open-source framework for large-scale, flexible evaluation of biomedical text mining systems
Source: J Biomed Discov Collab. 2008 Jan 29;3:1. doi: 10.1186/1747-5333-3-1 (PMC2276192; doi:10.1186/1747-5333-3-1)
Supplement: Additional file 1 — Evaluation platform source code. The additional file contains the software evaluation framework discussed in this paper. The most current release can be downloaded from SourceForge.net [1]. See the accompanying README file for instructions on installation and use. Also included in the distribution is a collection of UIMA wrappers for some commonly used BioNLP tools and annotated corpora. [file 1747-5333-3-1-S1.zip › bionlp_uima_repository-1.0.1/docs/api/src-html/edu/uchsc/ccp/uima/annotation/CCPDocumentSubSection_Type.html]

```
001    
002    /* First created by JCasGen Tue Aug 28 16:44:10 MDT 2007 */
003    package edu.uchsc.ccp.uima.annotation;
004    
005    import org.apache.uima.cas.Feature;
006    import org.apache.uima.cas.FeatureStructure;
007    import org.apache.uima.cas.Type;
008    import org.apache.uima.cas.impl.CASImpl;
009    import org.apache.uima.cas.impl.FSGenerator;
010    import org.apache.uima.cas.impl.FeatureImpl;
011    import org.apache.uima.cas.impl.TypeImpl;
012    import org.apache.uima.jcas.JCas;
013    import org.apache.uima.jcas.JCasRegistry;
014    
015    /** This annotation type is used to annotate document subsections.
016     * Updated by JCasGen Tue Aug 28 16:44:10 MDT 2007
017     * @generated */
018    public class CCPDocumentSubSection_Type extends CCPDocumentAnnotation_Type {
019      /** @generated */
020      protected FSGenerator getFSGenerator() {return fsGenerator;}
021      /** @generated */
022      private final FSGenerator fsGenerator = 
023        new FSGenerator() {
024          public FeatureStructure createFS(int addr, CASImpl cas) {
025                             if (CCPDocumentSubSection_Type.this.useExistingInstance) {
026                               // Return eq fs instance if already created
027                         FeatureStructure fs = CCPDocumentSubSection_Type.this.jcas.getJfsFromCaddr(addr);
028                         if (null == fs) {
029                           fs = new CCPDocumentSubSection(addr, CCPDocumentSubSection_Type.this);
030                               CCPDocumentSubSection_Type.this.jcas.putJfsFromCaddr(addr, fs);
031                               return fs;
032                         }
033                         return fs;
034            } else return new CCPDocumentSubSection(addr, CCPDocumentSubSection_Type.this);
035              }
036        };
037      /** @generated */
038      public final static int typeIndexID = CCPDocumentSubSection.typeIndexID;
039      /** @generated 
040         @modifiable */
041      public final static boolean featOkTst = JCasRegistry.getFeatOkTst("edu.uchsc.ccp.uima.annotation.CCPDocumentSubSection");
042     
043      /** @generated */
044      final Feature casFeat_sectionName;
045      /** @generated */
046      final int     casFeatCode_sectionName;
047      /** @generated */ 
048      public String getSectionName(int addr) {
049            if (featOkTst && casFeat_sectionName == null)
050          jcas.throwFeatMissing("sectionName", "edu.uchsc.ccp.uima.annotation.CCPDocumentSubSection");
051        return ll_cas.ll_getStringValue(addr, casFeatCode_sectionName);
052      }
053      /** @generated */    
054      public void setSectionName(int addr, String v) {
055            if (featOkTst && casFeat_sectionName == null)
056          jcas.throwFeatMissing("sectionName", "edu.uchsc.ccp.uima.annotation.CCPDocumentSubSection");
057        ll_cas.ll_setStringValue(addr, casFeatCode_sectionName, v);}
058        
059      
060    
061    
062    
063      /** initialize variables to correspond with Cas Type and Features
064            * @generated */
065      public CCPDocumentSubSection_Type(JCas jcas, Type casType) {
066        super(jcas, casType);
067        casImpl.getFSClassRegistry().addGeneratorForType((TypeImpl)this.casType, getFSGenerator());
068    
069     
070        casFeat_sectionName = jcas.getRequiredFeatureDE(casType, "sectionName", "uima.cas.String", featOkTst);
071        casFeatCode_sectionName  = (null == casFeat_sectionName) ? JCas.INVALID_FEATURE_CODE : ((FeatureImpl)casFeat_sectionName).getCode();
072    
073      }
074    }
075    
076    
077    
078
```
